# Editorial Notes

**Published:** 1937

**Authors:** 


### Editorial Notes

**National Health Campaign.**

At a well-attended meeting held in the Colston Hall on Thursday, December 16th, Sir Kingsley Wood, Minister of Health, and Mr. Arthur Greenwood, who held the same office from 1929 to 1931, spoke in support of the National Health Campaign. The aim of this campaign as set forth by the principal speakers is to produce a physically fitter nation by inducing every individual to do all in their own power to make him or herself healthier, aided by the medical profession and the many State, municipal and voluntary agencies.

Speeches did not occupy the whole evening. A children's choir, directed by Mr. G. F. Lewis, sang surprisingly well: not perhaps attaining to the sweetness and precision of some Welsh children's choirs, but none the less showing great musical ability and life. There were also excellent "Keep Fit" displays by the Bristol Women Teachers' and the Bristol Schoolmasters' Physical Training Associations.

Sir Kingsley Wood spoke of the Health Campaign as a fight for better health and better conditions, in which we should need to use the whole armoury which modern science and knowledge have given us, better housing, improved nutrition, more care for the mothers and the children, more open spaces and recreation grounds and opportunities for exercise and physical training. But he was careful to explain that his idea of improving the physique of the nation was not the setting up of a course of physical jerks. He invited the co-operation of the general practitioners and of the voluntary institutions with the State and municipal services, paying several compliments to Bristol's record in social and medical progress. He referred appreciatively to the recent amalgamation of the Royal Infirmary and General Hospital, expressing his hope that this would facilitate still closer co-operation between the municipal hospitals, the voluntary hospitals and the University.

Mr. Greenwood, who spoke immediately after Sir Kingsley Wood, was untrammelled by the responsibilities of office and came nearer to the core of the matter. "These physical displays," he said, "necessary as they may be, are no alternative to a proper system of social services. . . . To make this a great nation you must see that there is no under-nourishment, and no social conditions which are injurious to healthy and inspiring growth." This latter phrase of Mr. Greenwood's sums up all that need be said in this present campaign. His words must be implemented by action. If we secure good food, good fresh air and good rest for our children they will dance and sing without orders from higher authority.

**New Medical Journals.**

Two new medical journals have made their first appearance this autumn. The *Irish Medical Journal* is the official organ of the Irish Medical Union, the association of medical practitioners in Eire (formerly known as the Irish Free State), which is the counterpart of the British Medical Association. This weekly journal is devoted to recording the proceedings of the Medical Union, and articles of medical, medico-political and general interest. It is modelled on the journals of other similar associations such as the American, Canadian, Australian and Indian. An official publication of this nature is essential to the life and activity of the Irish Medical Union. We have no doubt that the *Irish Medical Journal* will fully maintain the long tradition of first-rate medical journalism of which Ireland may well be proud. *Medicine of To-day and To-morrow* voices the opinions and views of the Socialist Medical Association. In the Editorial of the first number it is claimed that new ground is being broken. The journal is intended to help in replanning our medical system. Mr. Attlee, Leader of the Parliamentary Labour Party, sending his greetings to the new venture, says "it will be a great advantage to have a journal which will show the developments of planned medicine all over the world." In the first number there are two important articles: "The Municipal Hospitals of London," by Somerville Hastings, M.D., F.R.C.S., and "The Course of the Malnutrition Controversy," by Professor Le Gros Clark. *Medicine of To-day and To-morrow* is not designed solely for medical readers, and does not appear to compete with the *Lancet* and *British Medical Journal* in publishing scientific or clinical papers. If it continues as it has begun this journal, which is published monthly, will always provide interesting and informing reading. Even if some doubts may be felt whether medicine will benefit from the inclusion of politics, there can be no doubt that politics may be improved by an infusion of scientific methods. Perhaps *Medicine of To-day and To-morrow* will serve to blunt some of the asperities of political controversy.

**Revival of Territorial Medical Units in Bristol.**

SHORTLY after the War two medical units of the Territorial Army in Bristol were disbanded in the pursuit of a plan of economy and a hope that wars and rumours of wars would cease. Unfortunately that hope has been disappointed and rearmament is seen to be imperative. The War Office has ordered, and the Treasury has sanctioned, the formation of a Field Ambulance and a General Hospital in Bristol. The General Hospital will be revived under its former title of the Second Southern General Hospital, with Lieut.-Colonel R. C. Clarke, O.B.E., as Commanding Officer. His record begins in the days of the Volunteers, when he enlisted as a private in the Bristol Rifles (1st V.B. Gloucester Regiment); soon after the Territorial Force was formed Colonel Clarke received his commission as a subaltern in the same battalion (known as the 4th Gloucesters). Before the War he had transferred to the R.A.M.C., and remained with the battalion as its Medical Officer, proceeding to France in 1915 with the 48th (South Midland Division). During the War Captain Clarke (as he then was) was posted to No. 19 C.C.S. in charge of the Medical Division, and acted on many occasions as Consulting Physician to the Third Army. His war services were rewarded by the O.B.E. (Military Division).

On demobilization he returned to his former appointment of Medical Officer to the 4th Gloucesters, which he has held until gazetted to raise and command the Second Southern General Hospital. Colonel Clarke is by a great number of years the senior member of the 48th Division, and his appointment to this important command is a matter of general gratification.

The Field Ambulance is to be numbered the 144th in the Territorial Army. This was the number which belonged to the 3rd South Midland Field Ambulance in the War, but it was by the latter title it was generally known. The new field ambulance, like its predecessor, is part of the 144th Brigade in the 48th Division. Lieut.-Colonel W. A. Jackman, F.R.C.S., has been gazetted to take command. He served as a combatant officer with the 6th Gloucesters in the early part of the War, then he was returned to his medical studies and, after qualifying, joined the R.A.M.C. (T.F.). As an R.A.M.C. officer he saw service in various theatres of the War.

After the War he was again attached to the 6th Gloucesters as their Medical Officer, a post which he now vacates on promotion.

Those of us who recall the enthusiastic support which was given to Colonels Paul Bush and Arthur Prichard when these two units were originally raised in 1908 will wish that even greater success will attend the efforts of their successors. We hope, too, that the Drum-Major's staff, presented by the Bristol Medico-Chirurgical Society to the 3rd South Midland Field Ambulance on 9th July, 1909, will be seen at the head of the band once more, if bands are included in the establishment of field ambulances in these days. An interesting account of the early days of the 3rd South Midland Field Ambulance, written for this *Journal* by Lieut.-Colonel A. W. Prichard, was published in Vol. XXXI. (1913), p. 159.

